# Alignment of PrEP adherence and HIV exposure risk among pregnant and postpartum women in Lilongwe, Malawi

**DOI:** 10.1371/journal.pone.0335429

**Published:** 2025-10-23

**Authors:** Anna M. Leone, Friday Saidi, Lauren A. Graybill, Qinghua Li, Twambilile Phanga, Feng-Chang Lin, Twaambo E. Hamoonga, K. Rivet Amico, Wilbroad Mutale, Benjamin H. Chi

**Affiliations:** 1 Department of Obstetrics & Gynecology, University of North Carolina at Chapel Hill, Chapel Hill, North Carolina, United States of America; 2 UNC Project Malawi, Lilongwe, Malawi; 3 Institute for Global Health and Infectious Diseases, University of North Carolina at Chapel Hill, Chapel Hill, North Carolina, United States of America; 4 Gillings School of Public Health, University of North Carolina at Chapel Hill, Chapel Hill, North Carolina, United States of America; 5 Department of Population Studies and Global Health, University of Zambia, Lusaka, Zambia; 6 Department of Health Behavior and Health Equity, University of Michigan, Ann Arbor, Michigan, United States of America; 7 Department of Health Policy and Systems, University of Zambia, Lusaka, Zambia; Innovative Aid, CANADA

## Abstract

**Objective:**

When measured continuously, adherence to HIV pre-exposure prophylaxis (PrEP) is consistently low in studies of pregnant and postpartum women. We investigated how PrEP adherence aligned with HIV exposure risk.

**Methods:**

We conducted a trial of a PrEP adherence support intervention in Lilongwe, Malawi. Pregnant women who met eligibility criteria for PrEP had visits at three and six months following enrollment. At each visit, HIV exposure risk was categorized as low or moderate/high (i.e., higher) risk based on an algorithm. PrEP adherence was measured via tenofovir concentrations, with functional adherence defined at levels consistent with ≥4 doses/week. HIV exposure risk and PrEP adherence were classified as either aligned (i.e., higher HIV risk/PrEP adherence, low HIV risk/PrEP non-adherence) or not aligned (i.e., higher HIV risk/PrEP non-adherence, low HIV risk/PrEP adherence). Probability differences (PD) were used to estimate the effect of the PrEP adherence intervention on aligned PrEP adherence.

**Results:**

164 women were included in the analysis. HIV exposure risk was higher for 81 participants (49%) at three months and 89 (54%) at six months. PrEP adherence was low at three months (34%) and at six months (29%). Aligned PrEP adherence was observed in 89 (54%) participants at three months and 83 (51%) at six months. 62% at higher HIV exposure risk were not aligned at month three, which increased to 68% at month six. The probability of aligned PrEP adherence was greater among those randomized to the intervention than those receiving standard of care at three months (PD:15.7%; 95%CI:0.8%, 30.6%). This was also evident in analyses that considered women with high HIV risk but low adherence.

**Conclusion:**

Alignment of PrEP adherence with HIV exposure risk was dynamic. PrEP adherence should be considered in the context of evolving HIV exposure risk during pregnancy and postpartum, with greater emphasis on periods of elevated HIV risk exposure.

## Introduction

Women in sub-Saharan Africa (SSA) experience a high risk of HIV acquisition when pregnant and postpartum [1–3]. Alongside the risks for long-term morbidity and mortality, new maternal HIV acquisitions account for nearly 30% of vertical transmission cases in SSA [1–4]. Pre-exposure prophylaxis (PrEP) in the form of daily oral emtricitabine/tenofovir disoproxil fumarate (FTC/TDF) has been shown to safely and effectively prevent new HIV acquisition [5,6]. However, adherence to these daily regimens remains a challenge. In studies conducted among pregnant and postpartum women in SSA, adherence to PrEP has been low determined by patient return rates and drug level detection (ranging between 28% to 58% of each sample) and declines over time [7–9].

Oral PrEP adherence is typically defined by continuous daily dosing. However, perfect or near-perfect adherence to such regimens may not be practical for HIV prevention since HIV risk exposure changes over time [10–12]. Recent data suggest that such high adherence levels to daily oral PrEP may not be required to achieve a reduced risk of HIV acquisition [13,14]. The concept of “prevention-effective adherence” limits the measurement of PrEP usage in those periods where there is elevated risk for HIV acquisition [12,15]. The reframing of such adherence metrics may be important when assessing the effectiveness and population impact of PrEP. Velloza et al. for example, showed that higher HIV exposure risk was associated with high PrEP adherence among adolescent girls and young women in Kenya enrolled in the HPTN 082 trial [16]. In a qualitative exploration of experiences with prevention-effective adherence among sero-different couples in Uganda, 80% of interviewees maintained high adherence during periods of risk for HIV exposure [17]. To date, such prevention-effective adherence metrics have not been applied to studies of pregnant and postpartum women.

As part of the Tonse Padmodzi study, we conducted a randomized trial to evaluate a “status-neutral” adherence support intervention for antiretroviral therapy (for women living with HIV) and PrEP (for HIV-negative women eligible for PrEP according to national guidelines) in Malawi [18,19]. Among HIV-negative women, drug concentrations indicated that 32% of women at three months, and 28% of women at six months, were functionally adherent to PrEP in the month leading up to the study visit, with no differences noted between study arms [8]. It was unclear, however, how these adherence patterns changed in relation to HIV exposure risk, which may change over the course of pregnancy and postpartum. In this secondary analysis, we sought to characterize longitudinal alignment between HIV exposure risk and PrEP adherence and assess the effectiveness of our intervention using this nuanced adherence measure.

## Methods

### Study population, procedures, and intervention

The study was conducted at Bwaila District Hospital in Lilongwe, Malawi. The full protocol has been detailed elsewhere [19]. Briefly, pregnant women receiving antenatal care at the hospital were eligible for study screening if they were 18 years or older, had a negative HIV test in the last three months, were interested in taking daily oral PrEP, and were eligible to receive PrEP according to Malawi Ministry of Health guidelines (S1 Appendix) [20]. Pregnant women who reported a history of renal disease or a history of intimate partner violence or social harms were not eligible for enrollment. Those who met eligibility criteria provided written informed consent and a venous blood sample that was used to screen for HIV, hepatitis B, and elevated serum creatinine. Women with any of these conditions were excluded from enrollment. Following screening, eligible women responded to an interviewer-administered behavioral survey and were randomized 1:1 by permuted block design to receive either a combination adherence intervention or the standard of care.

The combination intervention package was made up of two parts: Integrated Next Step Counseling (iNSC) and an optional, self-identified adherence supporter [18]. Trained counselors provided iNSC at all study visits using a structured, motivational interviewing style approach to identify unmet needs related to well-being, sexual health and PrEP adherence and facilitate the development of plans to address these needs. The goal of the counseling was to tailor HIV prevention behavior with individual needs, including candid discussions about the woman’s desire to persist and adhere to PrEP. For women who identified an adherence supporter, the support person attended an orientation session within a month of enrollment during which they learned how to help reinforce adherence behaviors at home. The control group received standard of care antenatal counseling including education about HIV prevention and PrEP adherence.

Following enrollment, study visits were scheduled at one, three, and six months. At each study visit, participants were tested for HIV using rapid antibody tests. At the three- and six-month study visits, participants also provided a venous blood sample for renal monitoring and drug concentration measurement and responded to an interviewer-administered behavioral survey. At the six-month study visit, participants reported their pregnancy outcome. All participants were dispensed daily oral FTC/TDF for PrEP over the six-month follow-up period.

### Measures

**Fig 1:**
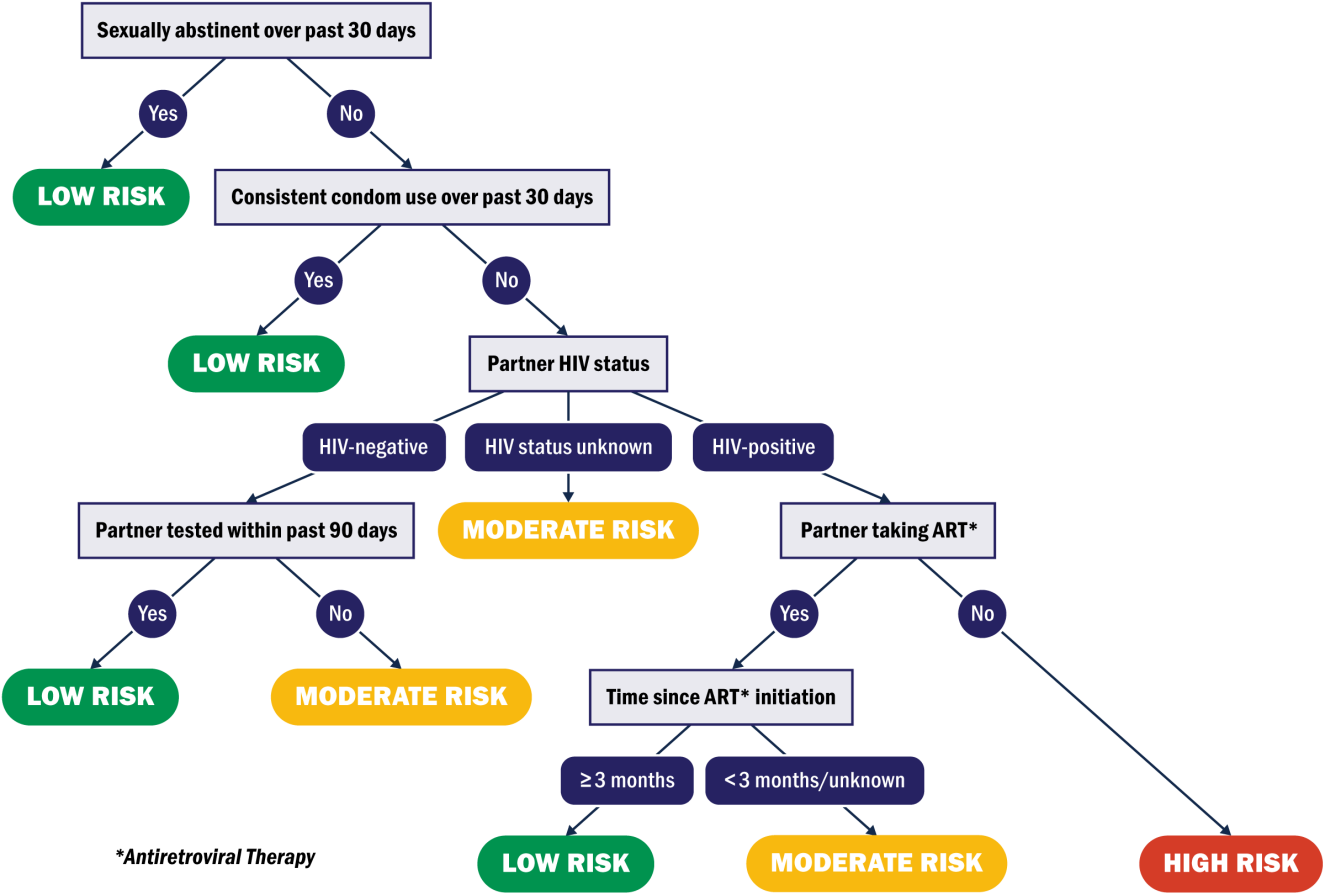
HIV exposure risk algorithm. HIV exposure risk algorithm based on available data from the Tonse Padmozi trial.

To measure HIV exposure risk, we analyzed data on sexual abstinence, condom use, partner HIV status, and partner ART use reported at three and six months. Using these variables, we created an algorithm to classify women as having a low, moderate, or high risk of acquiring HIV in the 30 days prior to the study visit (Fig 1). As few participants had a high risk of acquiring HIV, we dichotomized HIV risk into low risk and moderate/high risk for each study visit. The two groups are referred to as “low risk” and “higher risk” for the remainder of this report.

To assess PrEP adherence, we measured plasma and intracellular tenofovir concentrations in upper layer packed cells at three and six months. Using a published algorithm, we categorized PrEP adherence over the prior 28 days according to results from these two assays [21]. PrEP adherence scores ranged from 0 to 5 with a score of 0 representing a low number or no doses in the interval, and a score of 5 representing approximately daily dosing. We dichotomized this six-level PrEP adherence score to reflect “functional” adherence (scores 4 or 5), which was the equivalent of four or more doses per week. “Non-functional” adherence corresponded to less than four doses per week. Drug concentration testing was conducted at the University of North Carolina at Chapel Hill by the Clinical Pharmacology and Analytical Chemistry Core of the Center for AIDS Research.

To assess alignment of PrEP adherence and HIV exposure risk, we overlaid the two variables. Aligned PrEP adherence included two groups: (1) higher HIV exposure risk and PrEP adherent and (2) low HIV exposure risk and PrEP non-adherent. Conversely, non-alignment included the other two groups: (3) higher HIV risk and PrEP non-adherent and (4) low HIV risk and PrEP adherent.

We purposefully considered both non-alignment groups in our analysis. Those with higher HIV risk but low PrEP adherence (group 3 above) are clinically important because they are most likely to acquire HIV. Within prevention-effective adherence frameworks, this is the group most often prioritized for adherence support. In contrast, those with low HIV risk but high PrEP adherence (group 4 above) are relevant from the programmatic standpoint. Because of the favorable safety profile of daily oral FTC/TDF, their use of PrEP is unlikely to result in adverse events. However, from the perspective of service utilization, this group may be diverting resources from those in greater need. The higher rates of PrEP adherence in this subgroup may also overshadow important deficits in broader monitoring and evaluation efforts.

### Analytical approach

To characterize HIV exposure risk, PrEP adherence, and alignment of PrEP adherence with HIV exposure risk, we generated tornado plots. In each, participants were individually organized according to their time to delivery in order of gestational age at the time of enrollment because we hypothesized that HIV risk—and therefore PrEP adherence—could vary over the course of pregnancy and postpartum [22,23]. Time to delivery was measured by number of days prior to or following delivery, with time 0 signifying delivery. Within each tornado plot, participant follow-up time was divided into two periods: month 0–3 and month 3–6. Although assessments for HIV exposure risk and PrEP adherence only covered the four weeks prior to the study visit, we used these as proxies for the full three-month window. We created Sankey plots to show changes within the alignment categories for each time window.

To estimate the effect of the combination adherence intervention on aligned PrEP adherence, we compared the proportion of women with aligned PrEP adherence at three and six months between the intervention and control group using an estimated probability difference (PD) and a corresponding Wald 95% confidence interval (CI). Finally, we sought to understand the relative effect within each of the four groups according to HIV exposure risk and PrEP adherence. Because HIV risk was measured post-randomization and is expected to be influenced by the intervention, simple stratification by risk would not be valid, as risk lies on the causal pathway (i.e., risk functions as a mediator). Instead, we used a multinomial logistic regression model to estimate odds ratios (OR) and corresponding 95% CIs to compare the odds of each risk–adherence profile between intervention and control groups at each timepoint, with low HIV exposure risk/ PrEP non-adherent as the reference outcome category. The study population was restricted to participants with complete data on HIV exposure risk and PrEP adherence. All analyses were conducted using Windows SAS version 9.4 (SAS Institute, Cary, NC, USA) and R version 4.3.2.

### Ethical review

The study protocol was reviewed and approved by the National Health Services Research Committee (Lilongwe, Malawi) and the University of North Carolina at Chapel Hill Institutional Review Board (Chapel Hill, NC, USA).

## Results

From January 2020 to October 2022, 200 HIV-negative women were enrolled and randomized equally to intervention or control groups. Over the six-month follow-up period, 29 (14%) were lost to follow-up. An additional 7 (4%) participants had missing drug concentration data to assess PrEP adherence at three (n = 6) and six months (n = 1). Thus, the final analysis cohort comprised 164 women with complete data. Baseline characteristics of this analysis cohort are shown in Table 1. The mean age of participants was 25.5 years old with an average gestational age at time of enrollment of 25 weeks. Overall, 36% of participants perceived a “great chance” of acquiring HIV in the following 12 months with only 19% perceiving “no chance at all.” Twenty-one (13%) of participants had 4 or more lifetime sexual partners. Greater than 90% of participants were married to their primary partner. Approximately 1% had used condoms with their primary partner in the last 30 days. The 164 women in the final cohort differed from the 36 women not retained in the analytic sample by mean gestational age at time of enrollment and number of sexual intercourse acts in the past 30 days (S1 Table).

**Table 1:** Baseline characteristics of participants.

		Control Group (n = 81)	Intervention Group (n = 83)	Total (n = 164)
Age (y)	Mean (SD)	25.5 (5.3)	25.5 (5.5)	25.5 (5.4)
Gestational age at enrollment (weeks)	Mean (SD)	23.9 (9.2)	26.2 (8.3)	25.0 (8.8)
Gravidity	No prior pregnancies	15 (18.5%)	24 (28.9%)	39 (23.8%)
Number of living children	Mean (SD)	1.37 (1.24)	1.43 (1.39)	1.40 (1.31)
	No living children	21 (25.9%)	27 (32.5%)	48 (29.3%)
	1-2 living children	46 (56.8%)	38 (45.8%)	84 (51.2%)
	3 + living children	14 (17.3%)	18 (21.7%)	32 (19.5%)
Perceived risk of acquiring HIV in next 12 months	No chance at all	14 (17.3%)	18 (21.7%)	32 (19.5%)
	Small chance	19 (23.5%)	17 (20.5%)	36 (22.0%)
	Moderate chance	17 (21.0%)	19 (22.9%)	36 (22.0%)
	Great chance	31 (38.3%)	29 (34.9%)	60 (36.6%)
Number of lifetime sexual partners	1 lifetime sex partner	13 (16.0%)	25 (30.1%)	38 (23.2%)
	2-3 lifetime sex partners	52 (64.2%)	53 (63.9%)	105 (64.0%)
	4 + lifetime sex partners	16 (19.8%)	5 (6.0%)	21 (12.8%)
Relationship length (y) *	Mean (SD)	3.9 (4.6)	4.44 (4.6)	4.2 (4.6)
	< 1 year	21 (26.3%)	16 (20.0%)	37 (23.1%)
	1-4 years	38 (47.6%)	33 (41.3%)	71 (44.4%)
	5-9 years	10 (12.5%)	19 (23.8%)	29 (18.1%)
	10-14 years	7 (8.8%)	9 (11.3%)	16 (10.0%)
	15 + years	4 (5.0%)	3 (3.8%)	7 (4.4%)
Married to primary partner	Yes	74 (91.4%)	74 (89.2%)	148 (90.2%)
Number of sexual intercourse acts in the past 30 days *	Mean (SD)	6.6 (6.6)	8.1 (7.5)	7.4 (7.0)
Condom use with primary partner in the past 30 days ^	Mean (SD)	0.3 (0.8)	0.5 (1.4)	0.4 (1.2)
Primary Partner HIV Status *	HIV-negative	58 (72.5%)	60 (75.0%)	118 (73.8%)
	HIV-positive	5 (6.3%)	3 (3.8%)	8 (5.0%)
	Never tested	0 (0%)	3 (3.8%)	3 (1.9%)
	I don’t know	17 (21.3%)	14 (17.5%)	31 (19.4%)

* Among women who reported at least on sex partner in the past 3 months (n = 160).

^ Among women who reported at least one sex act in the past 30 days (n = 138).

**Fig 2:**
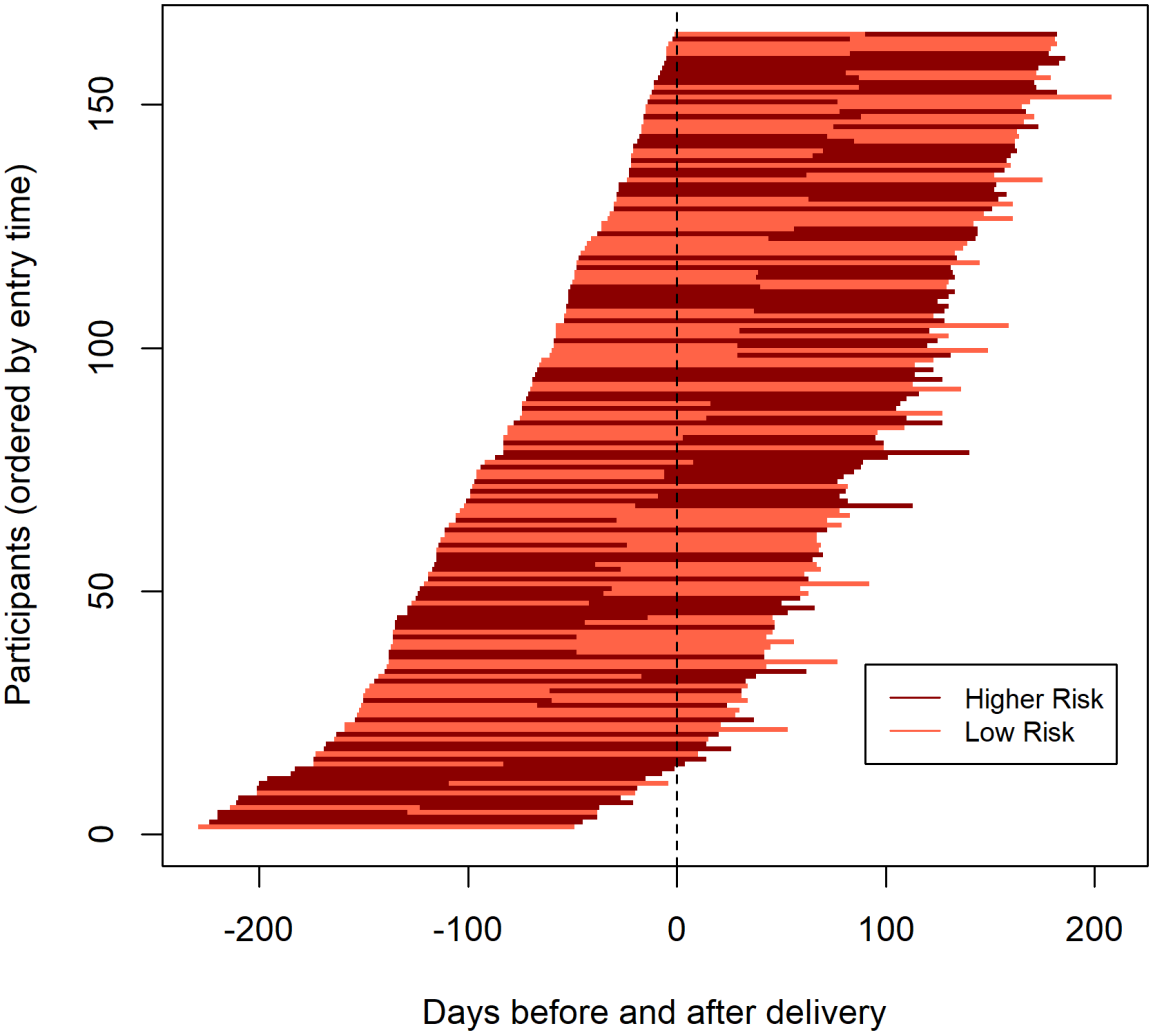
Tornado plot of participants’ individual HIV risk in relation to time of delivery.

**Fig 3:**
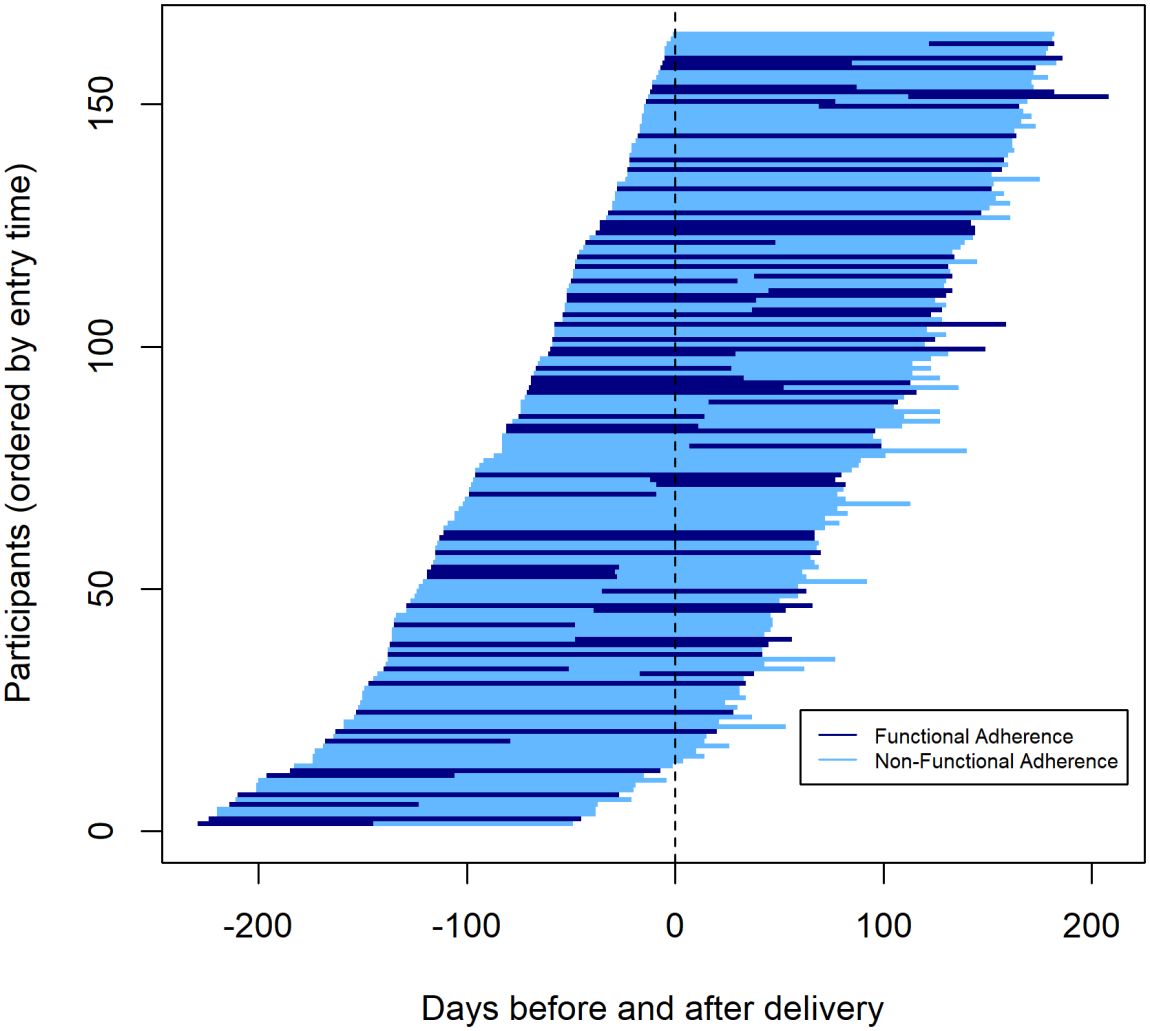
Tornado plot of participants’ PrEP adherence in relation to time of delivery.

Risk of HIV exposure over the two time periods (i.e., month 0–3 and month 3–6) is shown in Fig 2. At month 3, 83 (51%) participants were low risk and 81 (49%) were at higher risk for HIV exposure. At month 6, 75 (46%) participants were low risk and 89 (54%) were at higher risk. With our HIV risk assessment algorithm, the greatest contributor to increased HIV exposure risk was a partner who had not been tested for HIV in the past 90 days. When assessed by plasma and intracellular tenofovir concentrations, 58 (34%) participants had functional PrEP adherence at month 3 and 48 (29%) at month 6. Fig 3 shows trends in PrEP adherence over time. No clear trends in adherence to PrEP in relation to time to delivery were observed.

**Fig 4:**
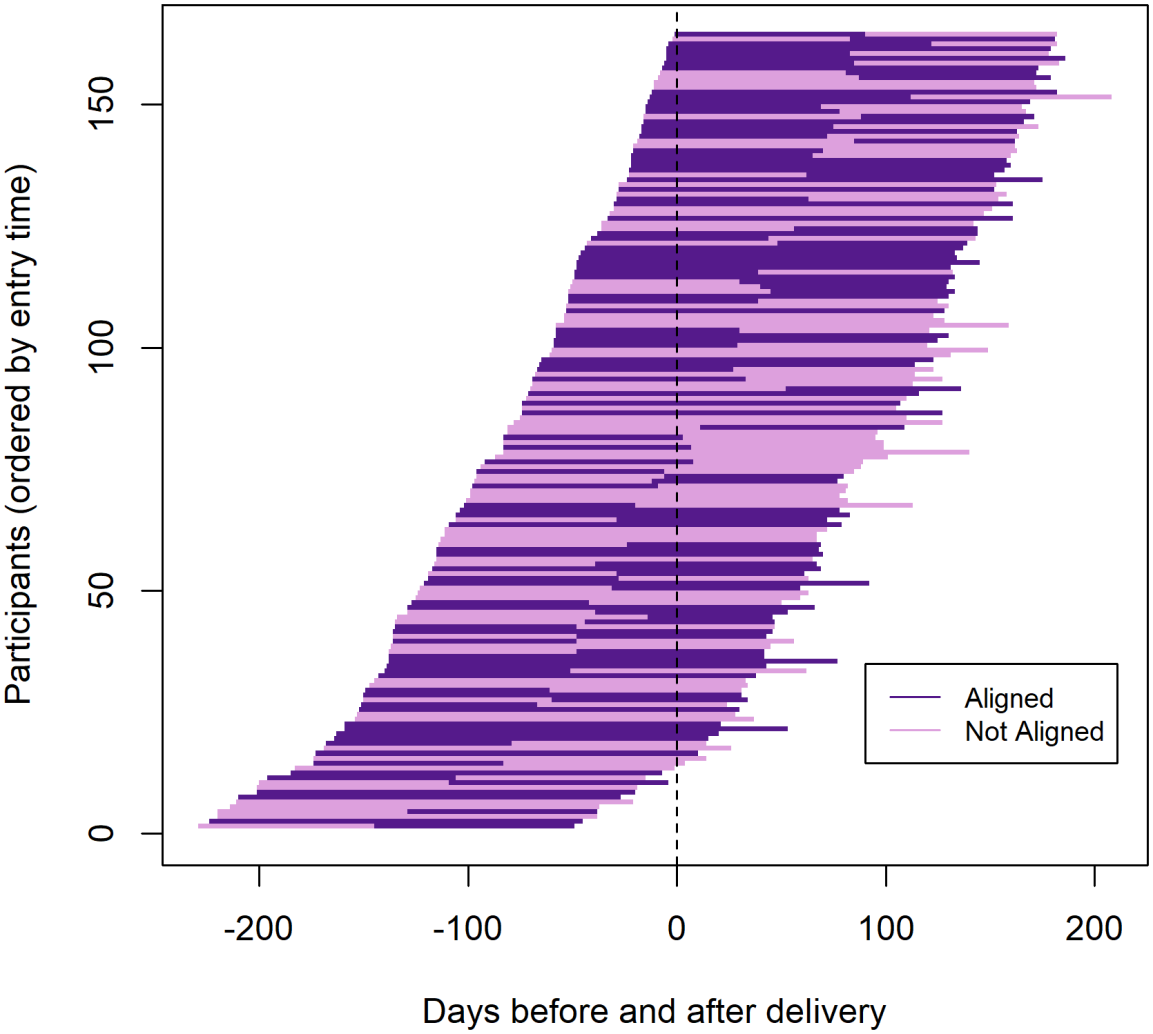
Tornado plot of participants’ alignment of PrEP adherence with HIV exposure risk in relation to time of delivery.

Approximately half of participants aligned PrEP adherence with their HIV exposure risk between months 0–3 (89; 54%) and between months 3–6 (83; 51%). Fig 4 illustrates aligned PrEP adherence by individual over the observation period. At each time period, the probability of alignment varied according to HIV exposure risk. Among women classified as having low HIV exposure risk, 70% (58 of 83) at three months and 73% (55 of 75) at six months were found to be aligned (i.e., PrEP non-adherent). In contrast, among women classified as having higher HIV exposure risk, 38% (31 of 81) at three months and 32% (28 of 89) at six months were found to be aligned (i.e., PrEP adherent). At three months, 62% (50 of 81) of women at higher HIV exposure risk were not aligned (i.e., PrEP non-adherent), and at six months, that percentage increased to 68% (61 of 89).

**Fig 5:**
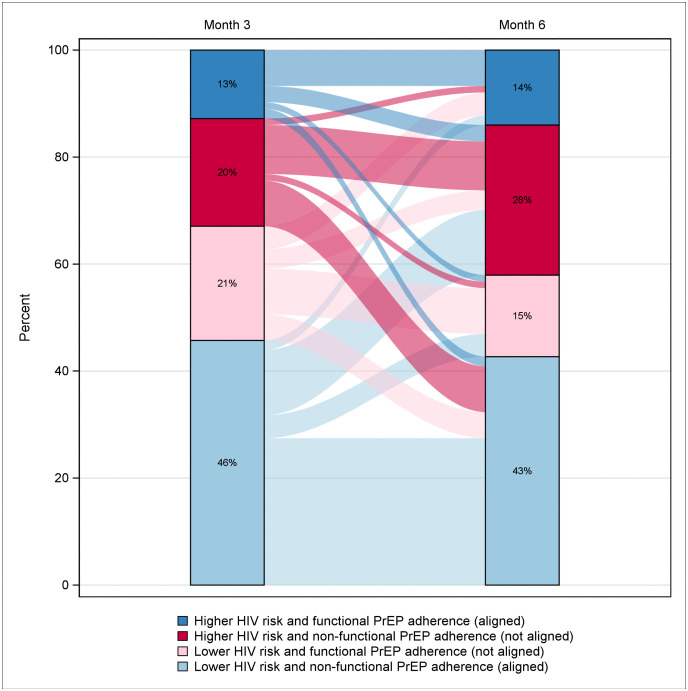
Sankey plot illustrating alignment of PrEP adherence and HIV exposure risk at month 3 and month 6.

Fig 5 shows how PrEP alignment changed in aggregate, including both aligned categories (higher HIV exposure risk/ PrEP adherent, low HIV exposure risk/ PrEP non-adherent) and non-aligned categories (higher HIV exposure risk/ PrEP non-adherent, low HIV exposure risk/ PrEP adherent). Movement was noted between groups. Notably, 30.7% (23 of 75) of women within the low HIV exposure risk and PrEP non- adherent group at month 3 shifted to higher HIV exposure risk at month 6. Eighty-seven percent of these women (20 of 23) were PrEP non-adherent at month 6. Only a small proportion of women who were higher HIV exposure risk and PrEP non-adherent at month 3, who remained higher risk at month 6, had drug concentrations consistent with functional PrEP adherence at month 6 (2 of 17; 11.8%).

With this outcome of aligned PrEP adherence, we evaluated the effect of the study’s adherence support intervention. The probability of aligned PrEP adherence was greater among women randomized to the intervention group than those in the control group at month 3 (66% vs 51%, PD: 15.7%, 95% CI: 0.8%, 30.6%; Table 2). The trend persisted at month 6, but the differences were attenuated and did not meet statistical significance (59% vs 54%, PD: 4.7%, 95% CI: −10.4%, 19.9%; Table 2).

**Table 2:** Effect of the Tonse Pamodzi combination adherence intervention on aligned PrEP adherence.

		n (%)	Unadjusted Risk Difference (95% CI)	Adjusted Risk Difference (95% CI)
Month 3	Intervention Group	55/83 (66.3%)	15.7% (0.8%, 30.6%)	15.9% (1.1%, 30.7%)
	Control Group	41/81 (50.6%)		
				
Month 6	Intervention Group	49/83 (59.0%)	4.7% (−10.4%, 19.9%)	6.3% (−8.8%, 21.3%)
	Control Group	44/81 (54.3%)		

Compared to participants randomized to the control group, those randomized to the intervention group had lower odds of being classified as higher HIV exposure risk/ PrEP non-adherent relative to low HIV risk/ PrEP non-adherent at three months (OR: 0.33, 95% CI: 0.14, 0.79) but not at six months (OR: 0.84, 95% CI: 0.40, 1.77). No differences were observed between intervention and control participants in the other risk–adherence profiles at three months or six months (Table 3).

**Table 3:** Effect of the Tonse Pamodzi combination adherence intervention on risk–adherence profiles.

	Intervention Group	Control Group	OR (95% CI)
	n (%)	n (%)	
**Month 3**			
Low risk/ Non-adherent	45/83 (54.2%)	30/81 (37.0%)	*ref*
Low risk/ Adherent	17/83 (20.5%)	18/81 (22.2%)	0.62 (0.28, 1.41)
Higher risk/ Non-adherent	11/83 (13.3%)	22/81 (27.2%)	0.33 (0.14, 0.79)
Higher risk/ Adherent	10/83 (12.1%)	11/81 (13.6%)	0.60 (0.23, 1.60)
**Month 6**			
Low risk/Non-adherent	38/83 (45.8%)	32/81 (39.5%)	*ref*
Low risk/Adherent	11/83 (13.3%)	14/81 (17.3%)	0.66 (0.26, 1.66)
Higher risk/Non-adherent	23/83 (27.7%)	23/81 (28.4%)	0.84 (0.40, 1.77)
Higher risk/Adherent	11/83 (13.3%)	12/81 (14.8%)	0.77 (0.30, 1.98)

## Discussion

In this study of pregnant and postpartum women, HIV exposure risk was high and appeared to increase slightly over time. PrEP adherence was low overall. Alignment of PrEP adherence with HIV exposure risk was dynamic and changed throughout the course of the study. At three months, the probability of aligned PrEP adherence was greater among women randomized to the adherence support intervention; however, this finding was no longer present by six months.

Prior studies have shown the difficulties in maintaining daily PrEP adherence even within limited timeframes such as pregnancy and postpartum. For example, Joseph Davey et al. described decreasing PrEP adherence in South Africa over the course of pregnancy and postpartum with 66% of women on PrEP at one month and 58% at three months [7]. In this study, the proportion of women who continued taking PrEP was lower during postpartum than during pregnancy [7]. In a study of pregnant women in Kenya, 58% of women persisted with PrEP adherence at nine months with 50% having quantifiable PrEP levels [9]. Multiple factors contribute to continued PrEP adherence—including partner behaviors, side effects, and, importantly, perceptions of HIV exposure risk—as shown in Zimbabwe [15] and South Africa [7].

We considered PrEP adherence in the context of HIV exposure risk. While such outcomes have been studied in other populations [16,17,24], to our knowledge, this is among the first in pregnant and postpartum women. In this analysis, we considered two forms of non-alignment (higher HIV exposure risk and PrEP non-adherent; low HIV risk and PrEP adherent); however, we recognize that the consequence for each scenario differs. For example, the scenario of higher HIV exposure risk and PrEP non-adherence carries greater consequence in terms of HIV acquisition. In fact, the measurement of prevention-effective adherence—i.e., the measurement of PrEP adherence during periods of elevated HIV exposure risk—emphasizes this type of alignment [12]. In contrast, for those at low HIV exposure risk, the main consequence of non-alignment is systems-related and unnecessary exposure to FTC/TDF. The over-utilization of PrEP can be an issue where drug availability is limited and, though individual safety risks are likely to be low given the generally favorable safety profile of daily oral FTC/TDF, complications may still occur [25]. There exists a balance between individual autonomy and public health goals that requires thoughtful exploration and discussion between the individual and their healthcare providers.

Examining the effect of the intervention on each risk-adherence profile presented a methodological challenge, since HIV risk lies on the causal pathway. To avoid conditioning on a post-randomization mediator, we employed a multinomial logistic regression model that simultaneously assessed intervention effects across all risk-adherence combinations. Compared to participants in the control group, those in the intervention group had lower odds of being classified as higher risk, non-adherent relative to low risk, non-adherent at three months but not at six months. This suggests that the intervention may have had an early effect on risk-adherence alignment, including among those with higher HIV risk but low PrEP adherence, but this dissipated over time. However, we caution against overinterpretation, since the sample sizes were relatively small and the estimates imprecise.

The study was originally designed to measure continual PrEP adherence over the course of follow-up. When our data were analyzed according to this traditional approach, there was no difference in adherence between the intervention and the control group at month 3 or month 6, and there were low PrEP adherence rates overall [8]. Based on these primary findings, we hypothesized that our study intervention—which integrated support for women’s decision making around sexual health, mental well-being, and drug adherence—may have promoted reconsideration of PrEP or self-discontinuation when risk was low. For example, women without HIV exposure risk may have been empowered to stop PrEP temporarily or for longer periods. Our findings here appear to support that notion. There appeared to be greater alignment between HIV exposure risk and PrEP adherence among those receiving the intervention at three months with the trend continuing at six months. For this and similar other behavioral interventions to support PrEP adherence, such nuanced metrics may be appropriate.

The algorithm used to characterize HIV exposure risk was created *post-hoc* based on available data and is not validated. Important variables, including viral suppression among partners with HIV, were not included because such information was not routinely available. Instead, we relied on proxy measures (e.g., time since partner ART initiation). In addition, the time window for some variables (e.g., partner HIV testing within the past 30 days) was not fully aligned with local clinical guidelines. However, we recognize the evolving nature of HIV exposure risk and, where necessary, opted for more conservative assumptions. Finally, while such algorithms provide valuable insights into potential HIV exposure risk, validated metrics—including descriptions of *perceived* HIV exposure risk—are needed.

Although there were many strengths to the current analysis, we note other limitations as well. First, the behaviors used to assess HIV exposure risk were self-reported and this could introduce bias. There may have been partner behaviors that were unknown to the participating woman and this could have affected the index woman’s HIV exposure risk. Given the inherent challenges to accurately collecting such information, approaches to triangulate data (e.g., partner enrollment, social network methods) could help to address otherwise underreported confounders. Second, our assessments of HIV exposure risk and PrEP adherence were based on the prior month; however, to simplify presentation, we considered them as proxy measures for the preceding three-month window. Although we felt such an approach was reasonable for the purposes of the current analysis, shorter and more frequent assessment windows would provide greater granularity into the evolving nature of HIV exposure risk and PrEP adherence. Third, we measured drug concentrations in two compartments where expected half-lives differ: plasma (i.e., 1–2 days) and intracellular tenofovir diphosphate concentrations in upper layer packed cells (i.e., several weeks). Newer assays—including from strands of hair—could provide data about FTC/TDF exposure over longer time windows; however, such specimens were not collected. Finally, the original study was designed to assess feasibility rather than clinical outcomes such as PrEP adherence. We recognize that, with its modest sample size, our estimates for the effect of the intervention on aligned PrEP adherence and HIV exposure risk lack precision. Nevertheless, they offer a nuanced approach to and insights on PrEP utilization. Future studies using such alignment outcomes, if properly powered, will be better positioned to show intervention effects.

In summary, we conducted an analysis to better characterize PrEP adherence in pregnant and postpartum women. This work highlights the evolving nature of HIV exposure risk during these periods and how that might affect PrEP adherence. Such contextual factors can have an important bearing on PrEP initiation, persistence, and adherence and should be considered in future studies.

## Supporting information

S1 Appendix Malawi Ministry of Health eligibility criteria for PrEP. (PDF)

S1 Table Baseline characteristics of participants retained and not retained in the analytic sample. (PDF)

S1 Checklist Inclusivity in global research. (DOCX)

## References

[1] Drake AL, Wagner A, Richardson B, John-Stewart G. Incident HIV during pregnancy and postpartum and risk of mother-to-child HIV transmission: a systematic review and meta-analysis. PLoS Med. 2014;11(2):e1001608. doi: 10.1371/journal.pmed.1001608 24586123 PMC3934828

[2] UNAIDS. New child infections due to gaps in prevention of vertical transmission; 2022. Available from: https://aidsinfo.unaids.org/

[3] Graybill LA, Kasaro M, Freeborn K, Walker JS, Poole C, Powers KA, et al. Incident HIV among pregnant and breast-feeding women in sub-Saharan Africa: a systematic review and meta-analysis. AIDS. 2020;34(5):761–76. doi: 10.1097/QAD.0000000000002487 32167990 PMC7275092

[4] WHO. WHO technical brief: preventing HIV during pregnancy and breastfeeding in the context of pre-exposure prophylaxis (PrEP). Geneva: World Health Organization; 2017.

[5] Joseph Davey DL, Pintye J, Baeten JM, Aldrovandi G, Baggaley R, Bekker L-G, et al. Emerging evidence from a systematic review of safety of pre-exposure prophylaxis for pregnant and postpartum women: where are we now and where are we heading? J Int AIDS Soc. 2020;23(1):e25426. doi: 10.1002/jia2.25426 31912985 PMC6948023

[6] Donnell D, Baeten JM, Bumpus NN, Brantley J, Bangsberg DR, Haberer JE, et al. HIV protective efficacy and correlates of tenofovir blood concentrations in a clinical trial of PrEP for HIV prevention. J Acquir Immune Defic Syndr. 2014;66(3):340–8. doi: 10.1097/QAI.0000000000000172 24784763 PMC4059553

[7] Joseph Davey DL, Mvududu R, Mashele N, Lesosky M, Khadka N, Bekker L-G, et al. Early pre-exposure prophylaxis (PrEP) initiation and continuation among pregnant and postpartum women in antenatal care in Cape Town, South Africa. J Int AIDS Soc. 2022;25(2):e25866. doi: 10.1002/jia2.25866 35138678 PMC8826542

[8] Chi BH, Saidi F, Graybill LA, Phanga T, Mollan KR, Amico KR. A patient-centered, combination intervention to support adherence to HIV pre-exposure prophylaxis during pregnancy and breastfeeding: a randomized pilot study in Malawi. J Acquir Immune Defic Syndr. 2023. 10.1097/QAI.0000000000003309 PMC10873086 37757844

[9] Pintye J, Kinuthia J, Abuna F, Anderson PL, Dettinger JC, Gomez L, et al. HIV pre-exposure prophylaxis initiation, persistence, and adherence during pregnancy through the postpartum period. AIDS. 2023;37(11):1725–37. doi: 10.1097/QAD.0000000000003617 37289583 PMC10527305

[10] Abaasa A, Hendrix C, Gandhi M, Anderson P, Kamali A, Kibengo F, et al. Utility of different adherence measures for PrEP: patterns and incremental value. AIDS Behav. 2018;22(4):1165–73. doi: 10.1007/s10461-017-1951-y 29090394 PMC5878836

[11] Hannaford A, Arens Y, Koenig H. Real-time monitoring and point-of-care testing: a review of the current landscape of PrEP adherence monitoring. Patient Prefer Adherence. 2021;15:259–69. doi: 10.2147/PPA.S248696 33574659 PMC7873020

[12] Haberer JE, Bangsberg DR, Baeten JM, Curran K, Koechlin F, Amico KR, et al. Defining success with HIV pre-exposure prophylaxis: a prevention-effective adherence paradigm. AIDS. 2015;29(11):1277–85. doi: 10.1097/QAD.0000000000000647 26103095 PMC4480436

[13] Marrazzo J, Tao L, Becker M, Leech AA, Taylor AW, Ussery F, et al. HIV preexposure prophylaxis with emtricitabine and tenofovir disoproxil fumarate among cisgender women. JAMA. 2024;331(11):930–7. doi: 10.1001/jama.2024.0464 38427359 PMC10951736

[14] Kiweewa FM, Ahmed K, Nair G, Cox S, Kintu A, Carter C, et al. Abstract Number 194. CROI; 2025.

[15] Gombe MM, Cakouros BE, Ncube G, Zwangobani N, Mareke P, Mkwamba A, et al. Key barriers and enablers associated with uptake and continuation of oral pre-exposure prophylaxis (PrEP) in the public sector in Zimbabwe: Qualitative perspectives of general population clients at high risk for HIV. PLoS One. 2020;15(1):e0227632. doi: 10.1371/journal.pone.0227632 31931514 PMC6957335

[16] Velloza J, Donnell D, Hosek S, Anderson PL, Chirenje ZM, Mgodi N, et al. Alignment of PrEP adherence with periods of HIV risk among adolescent girls and young women in South Africa and Zimbabwe: a secondary analysis of the HPTN 082 randomised controlled trial. Lancet HIV. 2022;9(10):e680–9. doi: 10.1016/S2352-3018(22)00195-3 36087612 PMC9530001

[17] Gilbert HN, Wyatt MA, Pisarski EE, Muwonge TR, Heffron R, Katabira ET, et al. PrEP discontinuation and prevention-effective adherence: experiences of PrEP users in Ugandan HIV serodiscordant couples. J Acquir Immune Defic Syndr. 2019;82(3):265–74. doi: 10.1097/QAI.0000000000002139 31609925 PMC6812551

[18] Hill LM, Saidi F, Freeborn K, Amico KR, Rosenberg NE, Maman S, et al. Tonse Pamodzi: developing a combination strategy to support adherence to antiretroviral therapy and HIV pre-exposure prophylaxis during pregnancy and breastfeeding. PLoS One. 2021;16(6):e0253280. doi: 10.1371/journal.pone.0253280 34170913 PMC8232532

[19] Saidi F, Mutale W, Freeborn K, Rosenberg NE, Graybill LA, Maman S, et al. Combination adherence strategy to support HIV antiretroviral therapy and pre-exposure prophylaxis adherence during pregnancy and breastfeeding: protocol for a pair of pilot randomised trials. BMJ Open. 2021;11(6):e046032. doi: 10.1136/bmjopen-2020-046032 34193491 PMC8246367

[20] Malawi Ministry of Health and Population. National guidelines for provision of oral pre-exposure prophylaxis for individuals at substantial risk of HIV in Malawi. 2020.

[21] Corneli AL, Deese J, Wang M, Taylor D, Ahmed K, Agot K, et al. FEM-PrEP: adherence patterns and factors associated with adherence to a daily oral study product for pre-exposure prophylaxis. J Acquir Immune Defic Syndr. 2014;66(3):324–31. doi: 10.1097/QAI.0000000000000158 25157647 PMC4059551

[22] Kinuthia J, Richardson BA, Drake AL, Matemo D, Unger JA, McClelland RS, et al. Sexual behavior and vaginal practices during pregnancy and postpartum: implications for HIV prevention strategies. J Acquir Immune Defic Syndr. 2017;74(2):142–9. doi: 10.1097/QAI.0000000000001225 27828872 PMC5357239

[23] Thomson KA, Hughes J, Baeten JM, John-Stewart G, Celum C, Cohen CR, et al. Increased risk of HIV acquisition among women throughout pregnancy and during the postpartum period: a prospective per-coital-act analysis among women with HIV-infected partners. J Infect Dis. 2018;218(1):16–25. doi: 10.1093/infdis/jiy113 29514254 PMC5989601

[24] Haberer JE, Kidoguchi L, Heffron R, Mugo N, Bukusi E, Katabira E, et al. Alignment of adherence and risk for HIV acquisition in a demonstration project of pre-exposure prophylaxis among HIV serodiscordant couples in Kenya and Uganda: a prospective analysis of prevention-effective adherence. J Int AIDS Soc. 2017;20(1):21842. doi: 10.7448/IAS.20.1.21842 28741331 PMC5577705

[25] Mofenson LM, Baggaley RC, Mameletzis I. Tenofovir disoproxil fumarate safety for women and their infants during pregnancy and breastfeeding. AIDS. 2017;31(2):213–32. doi: 10.1097/QAD.0000000000001313 27831952

