# Supplementary material for: Alignment of PrEP adherence and HIV exposure risk among pregnant and postpartum women in Lilongwe, Malawi
Source: PLoS One. 2025 Oct 23;20(10):e0335429. doi: 10.1371/journal.pone.0335429 (PMC12548898; doi:10.1371/journal.pone.0335429)
Supplement: S1 Appendix — (PDF) [file pone.0335429.s001.pdf]

S1 Appendix

Eligibility criteria for PrEP during the trial enrollment period

- Age 15 years or older
- HIV-negative test on day of PrEP initiation using the national HIV testing algorithm
- Client is at substantial risk of HIV infection
- Not found to have risk of acute HIV infection
- Client willingness to attend scheduled PrEP visits until 28 days after risk period
- No contraindication to use of TDF or 3TC
- Bodyweight of 30kg or greater
- Estimated glomerular filtration rate (eGFR) greater than or equal to 60ml/min
- No known renal diseases
- No diabetes mellitus

Reference: Malawi Ministry of Health and Population. National Guidelines for the Provision of Oral Pre-exposure Prophylaxis for Individuals at Substantial Risk of HIV in Malawi. 2020.
