# Supplementary material for: Alignment of PrEP adherence and HIV exposure risk among pregnant and postpartum women in Lilongwe, Malawi
Source: PLoS One. 2025 Oct 23;20(10):e0335429. doi: 10.1371/journal.pone.0335429 (PMC12548898; doi:10.1371/journal.pone.0335429)
Supplement: S1 Table — (PDF) [file pone.0335429.s002.pdf]

S2 Table. Baseline characteristics of participants retained and not retained in the analytic sample

		Retained (n=164)	Not retained (n=36)	p-value
Study arm	Intervention	81 (49.4%)	19 (52.8%)	0.7
Age (y)	< 20	5 (13.9%)	21 (12.8%)	
	20 to 24	16 (44.1%)	60 (36.6%)	
	25 to 29	9 (25.0%)	47 (28.7%)	0.9
	30 to 34	4 (11.1%)	24 (14.6%)	
	35 +	2 (5.6%)	12 (7.3%)	
Gestational age at enrollment (weeks)	Mean (SD)	25 (8.8)	29 (8.8)	0.03
Gravidity	No prior pregnancies	7 (19.4%)	39 (23.8%)	0.6
Number of living children	No living children	13 (36.1%)	48 (29.3%)	
	1-2 living children	17 (47.2%)	84 (51.2%)	0.7
	3+ living children	6 (16.7%)	32 (19.5%)	
Perceived risk of acquiring HIV in next 12 months	No chance at all	8 (22.2%)	32 (19.5%)	
	Small chance	6 (16.7%)	36 (22.0%)	0.8
	Moderate chance	10 (27.8%)	36 (22.0%)	
	Great chance	12 (33.3%)	60 (36.6%)	
Number of lifetime sexual partners	1 lifetime sex partner	11 (30.6%)	38 (23.2%)	
	2-3 lifetime sex partners	19 (52.8%)	105 (64.0%)	0.5
	4+ lifetime sex partners	6 (16.7%)	21 (12.8%)	
Relationship length (y) *	< 1 year	10 (27.8%)	37 (23.1%)	
	1-4 years	17 (47.2%)	71 (44.4%)	
	5-9 years	4 (11.1%)	29 (18.1%)	0.8
	10-14 years	4 (11.1%)	16 (10.0%)	
	15+ years	1 (2.8%)	7 (4.4%)	
Married to primary partner	Yes	35 (97.2%)	148 (92.5%)	0.3
Number of sexual intercourse acts in the past 30 days *	Mean (SD)	7.3 (1.0)	10.7 (7.8)	0.01
Condom use with primary partner in the past 30 days ^	Mean (SD)	0.4 (1.2)	0.1 (0.5)	0.2
Primary Partner HIV Status *	HIV-negative	118 (73.8%)	28 (77.8%)	
	HIV-positive	8 (5.0%)	2 (5.6%)	1.0
	Never tested	3 (1.9%)	0 (0.0%)	
	I don’t know	31 (19.4%)	6 (16.7%)	

* Among women who reported at least one sex partner in the past 3 months
^ Among women who reported at least one sex act in the past 30 days
